# Morphokinetic Analyses of Fishing Cat–Domestic Cat Interspecies Somatic Cell Nuclear Transfer Embryos Through A Time-Lapse System

**DOI:** 10.3390/ani15020148

**Published:** 2025-01-09

**Authors:** Hai-Jun Liu, Serena Jocelyn Wai Yin Oh, Nicole Liling Tay, Christina Yingyan Lim, Chia-Da Hsu, Delia Hwee Hoon Chua, Winnie Koon Lay Teo, Yuin-Han Loh, Soon Chye Ng

**Affiliations:** 1 Endangered Species Conservation via Assisted Reproduction (ESCAR) Lab, Institute of Molecular and Cell Biology (IMCB), Agency for Science, Technology and Research (A*STAR), 61 Biopolis Drive, Proteos, Singapore 138673, Singapore; liuhj67@126.com (H.-J.L.); sjowye@gmail.com (S.J.W.Y.O.); nicole.tay@mandai.com (N.L.T.); christina.lim@mandai.org.sg (C.Y.L.); klwteo@imcb.a-star.edu.sg (W.K.L.T.); yhloh@imcb.a-star.edu.sg (Y.-H.L.); 2 Mandai Wildlife Group, 80 Mandai Lake Road, Singapore 729826, Singapore; chiada.hsu@mandai.com (C.-D.H.); delia.chua@mandai.com (D.H.H.C.); 3 Cell Fate Engineering and Therapeutics Laboratory, Cell Biology and Therapies Division, Institute of Molecular and Cell Biology (IMCB), Agency for Science, Technology and Research (A*STAR), 61 Biopolis Drive, Proteos, Singapore 138673, Singapore; 4 Department of Biological Sciences, National University of Singapore, Singapore 117543, Singapore; 5 Department of Physiology, Yong Loo Lin School of Medicine, National University of Singapore, Singapore 117593, Singapore; 6 NUS Graduate School for Integrative Sciences and Engineering, National University of Singapore, 28 Medical Drive, Singapore 117456, Singapore; 7 Department of Obstetrics and Gynaecology, Yong Loo Lin School of Medicine, National University of Singapore, Singapore 119074, Singapore; 8 Sincere Healthcare Group, 8 Sinaran Drive, Singapore 307470, Singapore

**Keywords:** interspecies somatic cell nuclear transfer embryos, morphokinetic parameters, cleavage patterns, fishing cat

## Abstract

A time-lapse live embryo monitoring system provides a powerful approach to recording dynamic developmental events of cultured embryos in detail. By obtaining continuous short-interval images, blastocyst formation can be predicted and embryos can be selected. The objective of this study was to investigate the morphokinetic parameters of fishing cat–domestic cat interspecies somatic cell nuclear transfer (iSCNT) embryos from one-cell to blastocyst stages, and in particular, the cleavage patterns of the first division in iSCNT and IVF embryos, as these play a central role in euploidy. Domestic cat in vitro fertilization (IVF) embryos were set up as controls. The results show that morula and blastocyst development rates were significantly lower in the iSCNT embryos compared to their IVF counterparts. All earlier time points of embryonic development before the onset of blastulation in the iSCNT embryos were significantly delayed when compared with their IVF counterparts. In iSCNT, normal embryos (defined as those that developed to the blastocyst stage) took a longer time to reach the morula stage, and these morulas were more likely to undergo compaction, compared to their arrested embryo counterparts. Direct cleavage in the first division is a morphological aberration, and was seen with greater prevalence in iSCNT embryos than control IVF embryos; these aberrant embryos displayed a significantly lower blastocyst development rate than embryos that had undergone normal cleavage. In conclusion, the morphokinetic parameters of fishing cat–domestic cat iSCNT embryos at early stages could be used to predict their potential for development to the blastocyst stage. A time-lapse imaging system is potentially a powerful tool for selecting early embryos with developmental potential for transfer, and hence, for improving feline iSCNT efficiency.

## Simple Summary

Interspecies somatic cell nuclear transfer (iSCNT) is valuable for preserving endangered animals. The objective of this study was to investigate the morphokinetic parameters of fishing cat–domestic cat (iSCNT) embryos from one cell to blastocyst stages, and in particular, the cleavage patterns of the first division in iSCNT and IVF embryos, as these play a central role in euploidy. A time-lapse imaging system is potentially a powerful tool for selecting early embryos with developmental potential for transfer, and hence, for improving feline iSCNT efficiency.

## 1. Introduction

Compared to traditional embryo evaluation approaches, an automated time-lapse monitoring system avoids the need for embryos to be removed from the incubator, thus maintaining the culture in a stable and optimal environment at all times. This approach enables embryos to avoid the stress caused by exposure to adverse conditions such as fluctuations in temperature, gas concentration, pH, and humidity outside the incubator. Another significant advantage is that continuous images of the developing embryos can be acquired automatically at intervals of several minutes. These serial images provide detailed information for a more objective evaluation of the embryos through morphokinetic analysis.

Time-lapse technology is extensively used in human in vitro fertilization (IVF); 93% of blastocyst development stages can be accurately predicted based on three dynamic parameters related to cleavage division 48 h after fertilization [1]. In several retrospective cohort studies, using a time-lapse system for culturing and selecting embryos significantly improved the rates of pregnancy and/or implantation after transfer, compared to using a traditional incubator [2,3,4].

Now, time-lapse technology has extended its applications to bovine, sheep, and domestic cat IVF. The effects of abnormal cleavage patterns on embryonic developmental competence in reaching the blastocyst stage in IVF embryos have been investigated in cats [5,6], cattle [7], and sheep [8]. Morphokinetic parameters of IVF embryos and their relationships to in vitro developmental potential have also been assessed in cats and sheep [6,8].

So far, only three studies have explored somatic cell nuclear transfer (SCNT) embryonic development through a time-lapse system. Two of these studies mainly focused on recording the timing of SCNT embryos reaching different developmental stages in cattle [9] and pigs [10]. In mice, early morphokinetic parameters were identified to predict the blastocyst formation of SCNT embryos [11].

Interspecies SCNT (iSCNT) is valuable for preserving endangered species of animals [12,13]. Assisted reproductive technologies (ARTs), especially iSCNT, have proven its application potential for preserving endangered feline species. Utilizing enucleated domestic cat oocytes as recipients, iSCNT embryos were reconstructed in some endangered felids, such as the African wildcat [14], sand cat [15], leopard cat [16], marbled cat, flat-headed cat [17], and kodkod [18]. However, the development capability of reconstructed embryos in vitro and/or in vivo was low. The taxonomic distance between species from which donor nuclei and recipient oocytes come may negatively affect iSCNT efficiency. To date, live iSCNT offspring in felids were successfully produced only in African wildcats and sand cats, both of which belong to the same genus as the domestic cat [14,15]. However, intergeneric SCNT embryos of the leopard cat–domestic cat or flat-headed cat–domestic cat were unable to develop to term, despite successful implantation [16,17].

The fishing cat (*Prionailurus viverrinus*) is a unique wetland felid, endemic to South and Southeast Asia. Classified as vulnerable [19], wild populations are most threatened by habitat degradation and fragmentation. For conservation purposes, it is worth attempting to propagate fishing cat individuals through iSCNT technology. However, as with the leopard cat and flat-headed cat, the taxonomic relationship between fishing cats and domestic cats is extrageneric, so intergeneric SCNT between these two species is expected to be challenging.

In several human studies, time-lapse imaging revealed that intracytoplasmic sperm injection (ICSI) embryos with abnormal first cleavage had significantly reduced blastocyst development potential and implantation rates compared to normal cleavage embryos [20,21,22,23]. Such information is valuable for selecting embryos for transfer. In order to reduce the adverse effects caused by long-term in vitro culture, SCNT embryos are often transferred into surrogate oviducts at the early one- to four-cell stages in most species, such as in pigs, goats, sheep, and cats. SCNT outcomes could potentially be improved by the selection of normal cleavage embryos over embryos that underwent an abnormal first cleavage.

To date, morphokenetics parameters of interspecies SCNT embryos as well as feline SCNT embryos have not been investigated.

The three objectives of this current study were (1) to use a time-lapse imaging system to acquire detailed morphokinetic characteristics of fishing cat-domestic cat iSCNT and domestic cat IVF embryos from one-cell to blastocyst stages, (2) to identify differences in morphokinetic parameters between successful fishing cat–domestic cat iSCNT blastocysts and their arrested counterparts as well as domestic cat IVF embryos, and (3) to investigate the relationship between the cleavage patterns of first division in fishing cat–domestic cat iSCNT embryos and their subsequent blastocyst developmental ability.

## 2. Materials and Methods

### 2.1. Chemicals

All chemicals were purchased from Sigma-Aldrich (St. Louis, MO, USA) unless specified.

### 2.2. In Vitro Maturation (IVM) of Oocytes

Domestic cat ovaries were donated by local veterinary clinics, and transported to the laboratory in PBS at 4 °C, where they were stored at 4 °C for 12–24 h. Cumulus–oocyte complexes (COCs) were obtained through the dissection of the ovarian cortex into TCM 199-Hepes medium (Gibco, Grand Island, NY, USA) with 4 mg/mL BSA. Oocytes were classified as previously described [24]. Selected oocytes with homogeneous ooplasm and compact cumulus cells were cultured for in vitro maturation (IVM) in four-well plates (NUNC; Thermo Fisher Scientific, Waltham, MA, USA) in TCM-199 supplemented with 4 mg/mL BSA, 1 mM Sodium Pyruvate, 1.2 mM Cysteine, 2 mM L-Glutamine, 0.1 IU/mL FSH and LH, at 38.5 °C in a humidified atmosphere of 5% CO_2_, 5%O_2_, and 90% N_2_. After culturing for 24–30 h, cumulus cells were removed with 0.5% hyaluronidase. Oocytes with extruded first polar bodies were selected under a stereomicroscope SCNT or IVF.

### 2.3. Interspecies SCNT

#### 2.3.1. Donor Cells

Donor cells for iSCNT were derived from female fishing cat fibroblasts donated by the Mandai Wildlife Group cell biobank. Dividing cells of passages 6–8 at 80% confluency were dissociated with 0.25% trypsin (Gibco, Grand Island, NY, USA).

#### 2.3.2. Nuclear Transfer

Nuclear transfer was performed as previously described [25,26]. Metaphase II oocytes preincubated with Hoechst 33342 were enucleated by exposure to UV light. Donor cells were introduced into the perivitelline space of enucleated oocytes. Two pulses (2.0 KV/cm, 25 μs) were applied for fusion of the reconstructed oocytes in fusion medium. The fused couplets were activated with 10 μg/mL Calcium Ionophore (A23187) for 5 min, then 10 μg/mL cycloheximide and 5 μg/mL cytochalasin B for 5 h.

### 2.4. In Vitro Fertilization

Domestic cat testes were donated by local veterinary clinics. Sperm from the epididymis were collected for IVF. Cauda epididymis was cut transversely into small pieces in TCM 199-Hepes (Gibco, Grand Island, NY, USA) with 4 mg/mL BSA, and incubated for 10 min. Collected sperm was stored at 4 °C overnight. The sperm was centrifuged with Sydney IVF Sperm Medium (Cook, Brisbane, Australia). After discarding the supernatant, 1 mL Sydney IVF Sperm Medium was added to the pellet. The sperm samples were incubated for 30 min at 38.5 °C and 5% CO_2_ for swim-up. The metaphase II oocytes were fertilized with the top motile sperm at a final concentration of 0.5~1 × 10^6^/mL for 15.5 h in Sydney IVF Fertilization Medium (Cook, Brisbane, Australia) at 38.5 °C in a humidified atmosphere of 5% CO_2_, 5% O_2_, and 90% N_2_.

### 2.5. In Vitro Embryo Culture and Time-Lapse Imaging

The reconstructed iSCNT embryos (after activation) or in vitro fertilized presumptive zygotes were placed individually in droplets of G-TL medium (Vitrolife, Göteburg, Sweden) in an Embryoscope^TM^ time-lapse incubator (ES-D2-944; Vitrolife, Göteburg, Sweden) at 38.5 °C with 5% CO_2_, 5%O_2_, 90% N_2_ for 188-215 h (iSCNT embryos) or 207 h (IVF embryos). Images of embryos were acquired every 5 min in five different focal planes.

For IVF embryos, “Time 0” was defined as the time when sperm was added to oocytes; for iSCNT embryos, “Time 0” was defined as the time when fused embryos were placed into the activation medium of cycloheximide and cytochalasin B in M199. Morphokenetic marks included the timing of division to the 2-, 3-, and 4-blastomere stages (t2, t3, t4); morula formation (first signs of morula, tM); compacted morula formation (first signs of compaction, tCM); early blastocyst (tSB); blastocyst (tB); expanded blastocyst (tEB); and hatching blastocyst (tHB) stages. The durations of the second cell cycle (cc2; t3 − t2) and synchronous divisions (s2; t4 − t3) were included.

Cleavage patterns in the first division were classified into normal cleavage and abnormal cleavage. Normal cleavage is defined as the division of a one-cell embryo into two blastomeres of equal sizes. Abnormal cleavage included direct cleavage and uneven cleavage. Direct cleavage is when a one-cell embryo divides directly into three or more blastomeres, obviating the 2-cell stage. Uneven cleavage is defined as the division of a one-cell embryo into two blastomeres with uneven size.

In iSCNT, normal embryos (NE) are defined as those that developed to the blastocyst stage, while arrested embryos (AE) are defined as those whose development was arrested at any stage before reaching the blastocyst stage.

### 2.6. Blastocyst Cell Counting

Total cell number was compared between iSCNT or IVF blastocysts. Blastocysts derived from iSCNT or IVF were incubated with 10 μg/mL Hoechst 33342 in TCM-199 medium for 15 min and imaged. Blastocyst cell number was counted with the aid of Image J version 1.46r software (NIH, Bethesda, MD, USA) applied to the images.

### 2.7. Data Analysis

The data were analyzed by Mann–Whitney test with GraphPad InStat 3.06 software (GraphPad, San Diego, CA, USA). Differences were defined as significant at *p* < 0.05.

## 3. Results

### 3.1. Comparison of In Vitro Developmental Capability of Fishing Cat-Domestic Cat iSCNT or Domestic Cat IVF Embryos

**Figure 1:**
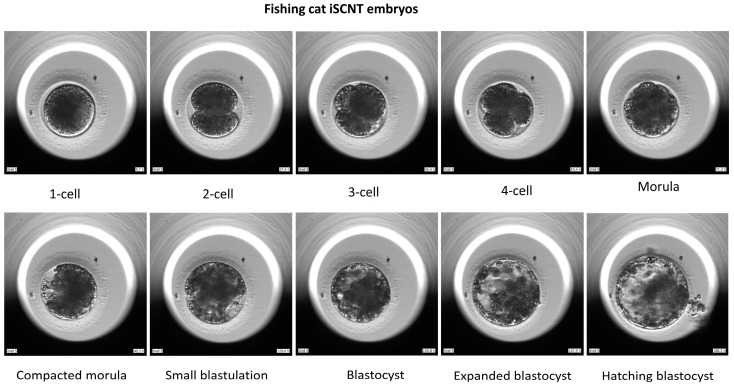
Development of fishing cat–domestic cat iSCNT embryos.

**Figure 2:**
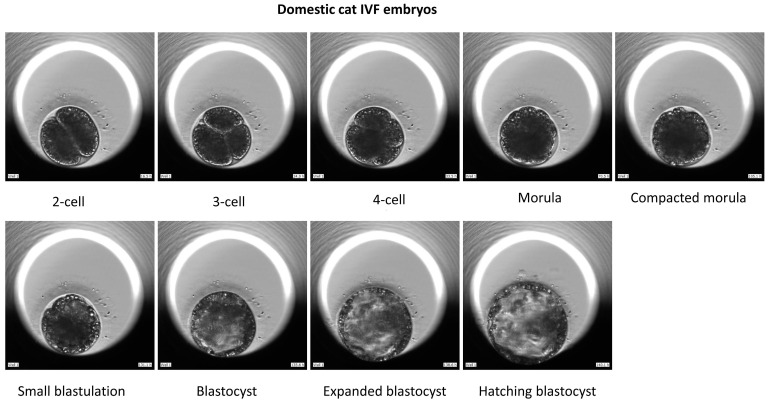
Development of domestic cat IVF embryos.

**Figure 3:**
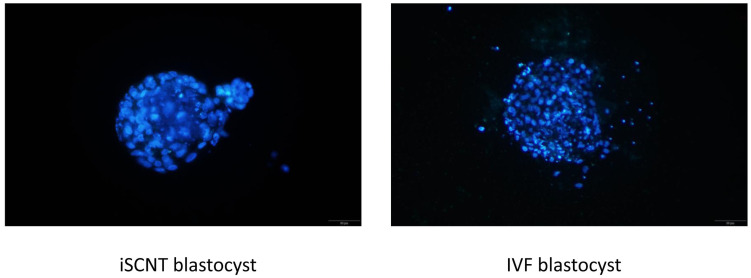
Blastocyst derived from iSCNT or IVF stained with Hoechst 33342.

Here, 216 oocytes collected from 36 domestic cat ovaries were selected for IVM, and 123 of them extruded first polar bodies. These matured oocytes were used for IVF or iSCNT. We investigated the differences in developmental capabilities between iSCNT embryos of fishing cat–domestic cat and domestic cat IVF embryos. The results show that the morula and blastocyst rates were significantly lower in iSCNT embryos compared with their IVF counterparts (*p* < 0.05) (Table 1). However, no differences were observed in the rates of cleavage, morula compaction, or hatching blastocyst, or in blastocyst cell numbers (Figure 1, Figure 2 and Figure 3; Videos S1 and S2).

### 3.2. Comparison of Morphokinetic Parameters of Fishing Cat–Domestic Cat iSCNT Embryos and Domestic Cat IVF Embryos

We compared the morphokinetic parameters of 8 fishing cat–domestic cat iSCNT embryos and 11 domestic cat IVF embryos from the first division to the blastocyst stage (Table 2). All the earlier stages of embryonic development before tSB (i.e., t2, t3, t4, tM, tCM) in iSCNT embryos were significantly delayed compared to their IVF counterparts (*p* < 0.05). However, all the later stages of embryonic development (i.e., tSB, tB, tEB, tHB) and the durations of cell cycles (cc2, s2) showed no significant difference between iSCNT and IVF embryos.

### 3.3. Comparison of Morphokinetic Parameters of Normal or Arrested Embryos Derived from Fishing Cat–Domestic Cat iSCNT

We compared the morphokinetic parameters of 8 normal and 24 arrested fishing cat–domestic cat iSCNT embryos from the first division to the compacted morula stage (Table 3). The mean tM and tCM were significantly longer in normal embryos compared to their arrested counterparts (*p* < 0.05). All earlier cleavage durations of embryonic development (i.e., t2, t3, t4), cc2, and s2 showed no significant differences between these two types of embryos.

### 3.4. Cleavage Patterns of the First Division in iSCNT and IVF Embryos and Their Relationship to Development to Blastocyst Stage

**Figure 4:**
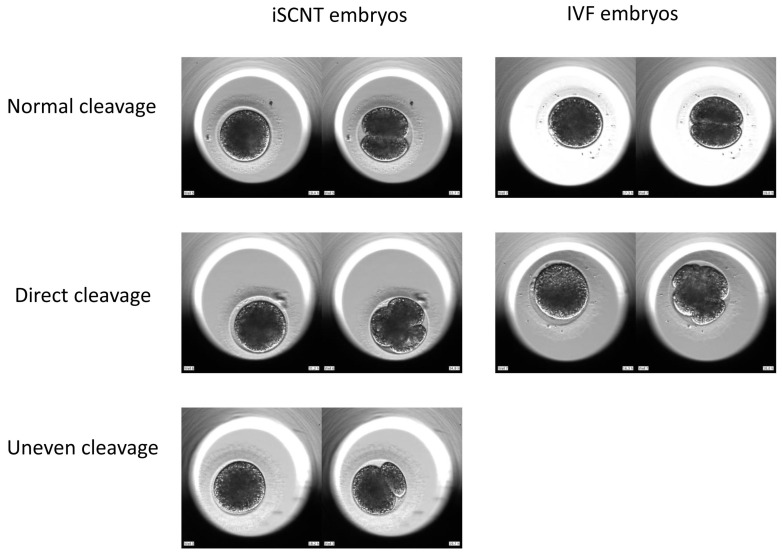
Cleavage patterns of iSCNT or IVF embryos at the first division.

We investigated the relationship between cleavage patterns of the first division and blastocyst development frequency in iSCNT and IVF embryos. In both iSCNT and IVF embryos, morphologically aberrant direct cleavage at the first division occurred at high proportions (14/32, 43.8%; 5/14, 35.7%; respectively). Compared to normal cleavage embryos, direct cleavage embryos displayed significantly lower blastocyst development rates in iSCNT embryos (43.8% vs. 7.7%; *p* < 0.05). Also, significantly more IVF embryos from direct cleavage reached the blastocyst stage than their iSCNT counterparts (60% vs. 7.7%; *p* < 0.05) (Table 4; Figure 4; Videos S3–S7).

## 4. Discussion

An automated time-lapse monitoring system provides a powerful approach in which embryos can be cultured in a stable environment while recording their dynamic developmental events in detail by continuous short-interval imaging. The resulting information could be used to predict the success of blastocyst formation and to perform quality control to select embryos with greater development potential for transfer.

To our knowledge, this is the first study investigating the morphokenetics parameters of iSCNT embryos and feline SCNT embryos through time-lapse technology.

The present study has shown that fishing cat–domestic cat iSCNT embryos presented compromised abilities to develop to the blastocyst stage compared with their domestic cat IVF counterparts (25% vs. 78.6%). Concordant with our present results, previous studies have demonstrated that iSCNT embryos demonstrated significantly lower blastocyst formation rates than those of IVF embryos in flat-headed cat–domestic cat (8.4% vs. 61.4%) and kodkod–domestic cat (5.9% vs. 44.5%) embryos [17,18]. Recipient domestic cats failed to become pregnant after flat-headed cat iSCNT embryo transfer, but in contrast, all six recipients that received domestic cat IVF embryos established pregnancies, with three of them producing five live kittens [17].

More than two decades have elapsed since the birth of “Dolly” the sheep [27]. However, cloning efficiency remains as low as under 5% (live offsprings/reconstructed embryos) [28]. Incomplete epigenetic reprogramming such as aberrant H3K9me3, H3K27me3, and H3K4me3 could impede the preimplantation or postimplantation development of iSCNT embryos [29]. Such epigenetic modifications also play an important role in in mouse embryonic stem cell pluripotency [30,31]. Mimicking physiological reprogramming in oocytes and sperm in preparation for fertilization may be a more effective strategy to improve nuclear transfer efficiency, compared to the extensively applied epigenetic modification of donor cell reprogramming after nuclear transfer [32]. The overexpression of transcription factor double homeobox (Dux) greatly improves mouse SCNT efficiency [33], and the expression of 2C transcripts was Dux-dependent in 2-cell-like cells of mouse embryonic stem cells (mESCs) [34]. For iSCNT, the incompatibility between nuclear and mitochondrial DNA, mtDNA heteroplasmy, and genome activation (EGA) of reconstructed embryos might be additional barriers impairing embryonic development, compared to SCNT [35].

Our current results indicate that the fishing cat–domestic cat iSCNT developed slower than their domestic cat IVF counterparts at all the earlier embryonic development stages, from two-cell to compacted morula. We presumed that this difference might be caused by the process of the first cell cycle in iSCNT or IVF embryos. Our previous study found that mitotic metaphase and cleavage were first detected at 23 h and 24 h post-activation, respectively, in goat SCNT embryos; this occurred later than in IVF embryos (16 h and 21 h post-insemination, respectively) [25]. Mouse SCNT embryos also took a longer time from the second division to compaction compared to their ICSI counterparts [11]. The current results are consistent with these observations.

The current study found that the arrested fishing cat–domestic cat iSCNT embryos took a shorter time to develop to morula or compacted morula stages. This finding is consistent with that of arrested IVF ovine embryos, which developed to the morula stage faster than normal embryos (72.32 h vs. 104.45 h) [8]. It is possible that prolonged morula formation time might allow normal embryos to undergo the cell divisions necessary to reach the cell numbers required for further development to the blastocyst stage. Mouse arrested SCNT embryos displayed massive levels of dysregulation of genes associated with transcription pathways, compared to their normal counterparts as well as IVF embryos [36]. Our domestic cat IVF embryos developed to the morula stage at 59.05 ± 6.64 h, which is shorter than the 74 h in a previous report [37]. The average cell numbers of domestic cat IVF blastocysts were 121.5 ± 17.5, which are lower than the 156.7 and 204.4 in a previous report [38].

In this study, 50% of the iSCNT embryos showed abnormal cleavage patterns in the first division, indicating the high prevalence of abnormal cleavage in fishing cat–domestic cat iSCNT embryos. Most abnormal cleavages were direct cleavages (87.5%), with uneven cleavage making up the remaining type (12.5%). In contrast, 35.7% of domestic cat IVF embryos underwent direct cleavage. Direct cleavage in iSCNT embryos significantly decreased their developmental capability to the blastocyst stage, compared with normal cleavage embryos, as well as direct cleavage embryos in domestic cat IVF. In contrast with our results, mouse SCNT embryos never underwent direct cleavage, but experienced fragmentation as the most common abnormality [11]. This inconsistency may be due to species differences.

In accordance with our results, previous studies have demonstrated a high frequency of 46–48% of abnormal cleavage in domestic cat IVF embryos [5,6]. Abnormal cleavages include cytoplasmic fragmentation, vacuolation, direct cleavage, additional reverse cleavage, and asymmetry of blastomeres. In these studies, 36.6% of morphologically normal and 48.6% of morphologically abnormal embryos developed to the blastocyst stage [5]. Higher blastocyst and hatching blastocyst rates were obtained when culturing domestic cat IVF embryos in Primo Vision^®^ dishes compared with the classical individual and group culture systems [39].

Through time-lapse imaging, several human studies have showed that the blastocyst development and/or implantation potential of ICSI embryos is severely impaired if direct cleavage occurs at first cleavage after fertilization, compared to embryos that underwent normal cleavage [20,21,22,23]. Bovine IVF embryos exhibiting direct cleavage led to significantly lower blastocyst and hatched blastocyst rates, and decreased chromosomal euploidy rates, than that of their normal counterparts [7]. In sheep IVF, none of the embryos that underwent direct cleavage developed to the blastocyst stage [8].

To date, the cause of direct cleavage remains unclear. A possible explanation for this might be the formation of multipolar spindles and supernumerary centrioles caused by defective sperm [21,23,40]. Recent evidence suggests that PADI6 gene mutations might be related to the pre-implantation embryonic arrest caused by direct cleavage in human IVF/ICSI embryos [41]. Our previous study found multiple polar spindles at the first mitotic metaphase in goat SCNT embryos [25].

Direct cleavage can only be observed by time-lapse imaging, and not by traditional embryo evaluation methods. Due to the impaired blastocyst development and/or implantation potential of direct cleavage embryos, screening out these abnormal embryos for transfer at earlier stages could increase pregnancy and implantation rates for both IVF and SCNT embryos.

Further work that could be done includes investigating the cellular and molecular mechanism underlying direct cleavage patterns in fishing cat–domestic cat iSCNT embryos, and determining the in vivo developmental potential of fishing cat iSCNT embryos by transferring quality-controlled embryos into surrogate cats.

## 5. Conclusions

In conclusion, the morphokinetics parameters of fishing cat–domestic cat iSCNT embryos at early stages could be used to predict their potential to develop to the blastocyt stage. Time-lapse imaging systems are a powerful tool, whereby early embryos with greater developmental potential could be selected for transfer, thereby improving SCNT efficiency in felid and possibly other species.

## Figures and Tables

**Table 1:** In vitro development of fishing cat–domestic cat iSCNT embryos vs. domestic cat IVF embryos.

Group	No. of Reconstructed Couplets	No. of Fused Couplets (%)	No. of Inseminated Oocytes	No. of Cultured Embryos	No. of Cleavage (%)
iSCNT	72	49 (68.1)	---	44	32 (72.7)
IVF	---	---	16	16	14 (87.5)
	**No. of Morula** **(cleavage %)**	**No. of Compacted Morula (cleavage %)**	**No. of Blastocyst** **(cleavage %)**	**No. Hatching Blastocyst** **(blastocyst %)**	**Cell Number of Blastocyst**
iSCNT	13 (40.6) ^a^	12 (37.5)	8 (25) ^a^	4 (50)	88.3 + 9.4
IVF	14 (100) ^b^	12 (85.7)	11 (78.6) ^b^	10 (90.9)	121.5 ± 17.5

Note: Different superscripts within the same column (e.g., a, b) represent significant differences *(p* < 0.05).

**Table 2:** Morphokinetic characteristics of fishing cat–domestic cat iSCNT and domestic cat IVF embryos developed to the blastocyst stage.

Variables	iSCNT	No. of Embryos *	IVF	No. of Embryos **
t2	22.5 ± 3.53 ^a^	7	18.75 ± 3.42 ^b^	8
t3	30.61 ± 3.24 ^a^	7	23.3 ± 3.84 ^b^	8
t4	39.4 ± 7.6 ^a^	7	28.84 ± 3.98 ^b^	8
cc2	8.11 ± 3.6	7	4.55 ± 2.53	8
s2	8.79 ± 5.59	7	5.54 ± 2.93	8
tM	81.45 ± 6.03 ^a^	8	59.05 ± 6.64 ^b^	11
tCM	99 ± 6.42 ^a^	8	84.26 ± 7.94 ^b^	11
tSB	126.93 ± 9.32	8	125.4 ± 12.71	11
tB	130.3 ± 5.54	7	135.16 ± 10.17	11
tEB	140.87 ± 10.33	6	141.5 ± 12.46	11
tHB	148.2 ± 15.11	4	143.26 ± 6.61	10

Notes: Different superscripts within the same row (e.g., a, b) represent significant differences (*p* < 0.05). * one iSCNT embryo with direct cleavage was excluded from t2 to s2. ** three IVF embryos with direct cleavage were excluded from t2 to s2.

**Table 3:** Morphokinetic characteristics of normal or arrested embryos derived from fishing cat–domestic cat iSCNT.

Variables	Normal Emb	No. of Embryos *	Arrested Emb	No. of Embryos **
t2	22.5 ± 3.53	7	22.36 ± 6.15	7 ***
t3	30.61 ± 3.24	7	33.53 ± 7.81	7
t4	39.4 ± 7.6	7	39.5 ± 11.41	5
cc2	8.11 ± 3.6	7	11.17 ± 3.4	7
s2	9.5 ± 5.74	7	7.58 ± 7.23	5
tM	81.45 ± 6.03 ^a^	8	67.32 ± 2.61 ^b^	5
tCM	99 ± 6.42 ^a^	8	75.9 ± 1.86 ^b^	4

Notes: The final developmental stages of the 24 arrested iSCNT embryos were as follows: 2-cell, 3; 3-cell, 6; 4-cell, 3; 8-cell, 5; 16-cell, 2; morula, 1; compacted morula, 4. Different superscripts within the same row (e.g., a, b) represent significant differences (*p* < 0.05). * one normal iSCNT embryo with direct cleavage was excluded from t2 to s2. ** thirteen arrested iSCNT embryos with direct cleavage were excluded from t2 to s2. *** four 2-cell stage iSCNT were excluded, including three embryos arrested at the 2-cell stage and one 2-cell embryo cleaved directly into a 4-cell one.

**Table 4:** Cleavage patterns of the first division in iSCNT and IVF embryos and their relationship to development to the blastocyst stage.

	Group	iSCNT		IVF	
Cleavage Pattern		Cleaved	Blastocyst (Cleavage %)	Cleaved	Blastocyst (Cleavage %)
Normal cleavage		16	7 (43.8) ^a^	9	8 (88.9)
Direct cleavage		14	1 (7.7) ^b,c^	5	3 (60) ^d^
Uneven cleavage		2	0		
Total		32	8 (25.8)	14	11 (78.6)

Note: Different superscripts within the same column (e.g., a, b) or same row (e.g., c, d) represent significant differences *(p* < 0.05).

## Data Availability

The data for this study are included in the article and Supplementary Materials.

## Supplementary Materials

The following supporting information can be downloaded at: https://www.mdpi.com/article/10.3390/ani15020148/s1, Video S1: iSCNT embryo development; Video S2: IVF embryo development; Video S3: Normal cleavage of iSCNT embryo; Video S4: Direct cleavage of iSCNT embryo; Video S5: Uneven cleavage of iSCNT embryo; Video S6: Normal cleavage of IVF embryo; Video S7: Direct cleavage of IVF embryo.
