# Supplementary material for: Morphokinetic Analyses of Fishing Cat–Domestic Cat Interspecies Somatic Cell Nuclear Transfer Embryos Through A Time-Lapse System
Source: Animals (Basel). 2025 Jan 9;15(2):148. doi: 10.3390/ani15020148 (PMC11758314; doi:10.3390/ani15020148)
Supplement: Supplementary file 1 [file animals-15-00148-s001.zip › Video legends.pdf]

Video legends:

Video S1: iSCNT embryo development

Video S2: IVF embryo development

Video S3: Normal cleavage of iSCNT embryo

Video S4: Direct cleavage of iSCNT embryo

Video S5: Uneven cleavage of iSCNT embryo

Video S6: Normal cleavage of IVF embryo

Video S7: Direct cleavage of IVF embryo
